# A monoclinic polymorph of di-μ-oxido-bis­({2-[2-(methyl­amino)ethyl­imino­methyl]phenolato-κ^3^ *N*,*N*′,*O*}oxidovanadium(V))

**DOI:** 10.1107/S160053680803328X

**Published:** 2008-11-13

**Authors:** Grzegorz Romanowski, Michał Wera, Artur Sikorski

**Affiliations:** a University of Gdańsk, Faculty of Chemistry, Sobieskiego 18/19, 80-952 Gdańsk, Poland

## Abstract

A new monoclinic polymorph of the title compound, [V_2_(C_10_H_13_N_2_O)_2_O_4_], which is a centrosymmetric dimer, crystallizes in space group *P*2_1_/*c*, whereas the previously known polymorph crystallizes in the ortho­rhom­bic space group *Pbca* [Mokry & Carrano (1993 ▶). *Inorg. Chem.* **32**, 6119–6121]. Each V^V^ atom is six-coordinated by one oxide group, two N atoms and one O atom from the Schiff base ligand, and by two additional bridging O atoms. The two methyl­ene groups are each disordered over two sites, with occupancy factors of 0.776 (14) and 0.224 (14). In the crystal structure, there are C—H⋯O hydrogen bonds and C—H⋯π inter­actions between the dimers.

## Related literature

For general background, see: Butler & Walker (1993 ▶); Carter-Franklin *et al.* (2003 ▶); Eady (2003 ▶); Evangelou (2002 ▶); Mendz (1991 ▶); Rehder *et al.* (2003 ▶); Sakurai (2002 ▶). For related structures, see: Mokry & Carrano (1993 ▶); Rao *et al.* (1981 ▶); Romanowski *et al.* (2008 ▶); Root *et al.* (1993 ▶). For the synthesis, see: Kwiatkowski *et al.* (2003 ▶).
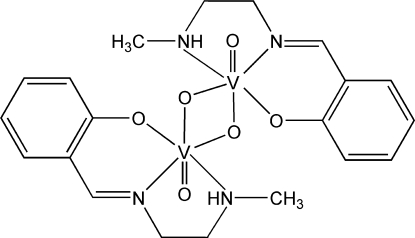


## Experimental

### 

#### Crystal data

[V_2_(C_10_H_13_N_2_O)_2_O_4_]
*M* *_r_* = 520.33
Monoclinic, 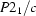

*a* = 6.6801 (2) Å
*b* = 11.9955 (6) Å
*c* = 13.8643 (7) Å
β = 92.156 (4)°
*V* = 1110.18 (9) Å^3^
*Z* = 2
Mo *K*α radiation
μ = 0.89 mm^−1^
*T* = 295 (2) K
0.6 × 0.1 × 0.1 mm

#### Data collection

Oxford Diffraction Ruby CCD diffractometer
Absorption correction: multi-scan (*CrysAlis RED*; Oxford Diffraction, 2006 ▶) *T*_min_ = 0.532, *T*_max_ = 0.915
6336 measured reflections
1960 independent reflections
1288 reflections with *I* > 2σ(*I*)
*R*_int_ = 0.050

#### Refinement

*R*[*F* ^2^ > 2σ(*F* ^2^)] = 0.044
*wR*(*F* ^2^) = 0.106
*S* = 0.90
1960 reflections
155 parameters
H-atom parameters constrained
Δρ_max_ = 0.36 e Å^−3^
Δρ_min_ = −0.39 e Å^−3^

### 

Data collection: *CrysAlis CCD* (Oxford Diffraction, 2006 ▶); cell refinement: *CrysAlis RED* (Oxford Diffraction, 2006 ▶); data reduction: *CrysAlis RED*; program(s) used to solve structure: *SHELXS97* (Sheldrick, 2008 ▶); program(s) used to refine structure: *SHELXL97* (Sheldrick, 2008 ▶); molecular graphics: *ORTEPII* (Johnson, 1976 ▶); software used to prepare material for publication: *SHELXL97* and *PLATON* (Spek, 2003 ▶).

## Supplementary Material

Crystal structure: contains datablocks I, global. DOI: 10.1107/S160053680803328X/hy2159sup1.cif

Structure factors: contains datablocks I. DOI: 10.1107/S160053680803328X/hy2159Isup2.hkl

Additional supplementary materials:  crystallographic information; 3D view; checkCIF report

## Figures and Tables

**Table 1:** Selected bond lengths (Å)

O7—V14	1.926 (2)
N9—V14	2.158 (3)
N12—V14	2.146 (3)
V14—O16	1.612 (2)
V14—O15	1.674 (2)
V14—O15^i^	2.316 (2)

Symmetry code: (i) 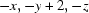.

**Table 2:** Hydrogen-bond geometry (Å, °)

*D*—H⋯*A*	*D*—H	H⋯*A*	*D*⋯*A*	*D*—H⋯*A*
C8—H8*A*⋯O16^ii^	0.93	2.60	3.520 (4)	170
C11*B*—H11*C*⋯*Cg*1^iii^^iii^	0.97	2.82	3.47 (2)	124

Symmetry codes: (ii) 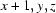; (iii) 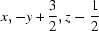. *Cg*1 is the centroid of the C1–C6 ring.

**Fig. 1.**
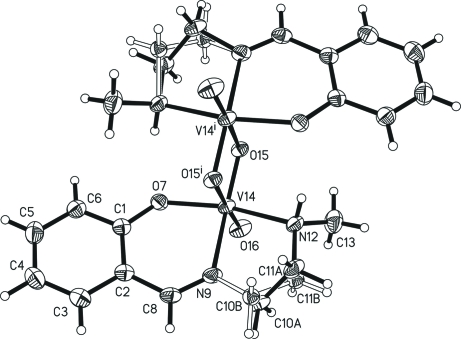
The molecular structure of the title compound, with displacement ellipsoids drawn at the 25% probability level. [Symmetry code: (i) -x, -y+2, -z.]

**Fig. 2.**
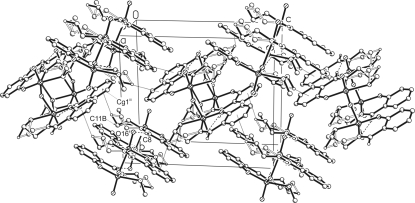
The arrangement of the molecules viewed approximately along the a-axis. The C—H···O hydrogen bonds are represented by dashed lines and the C—H···π interactions are represented by dotted lines. [Symmetry codes: (ii) 1+x, y, z; (iii) x, 3/2-y, -1/2+z.]

## supplementary crystallographic information

### Comment

In the past few decades, the interest in the coordination chemistry and biochemistry of vanadium compounds has increased due to their influence on biological systems, *viz*. in diabetes mellitus (Sakurai, 2002) and cancer treatment (Evangelou, 2002). Moreover, vanadium activity has been discovered in the inhibitory and promotory processes like nitrogenases (Eady, 2003), haloperoxidases (Butler & Walker, 1993; Carter-Franklin *et al.*, 2003; Rehder *et al.*, 2003), mutases and isomerases (Mendz, 1991).

The structure of the title compound was first reported in orthorhombic space group *Pbca* (Mokry & Carrano, 1993). Here we report the synthesis and structure of a new polymorph of the compound in space group *P*2_1_/*c*. We have described earlier the spectroscopic properties (IR, UV-Vis, ^1^H and ^51^V NMR) of this compound  (Kwiatkowski *et al.*, 2003). The half of the molecule, constituting the asymmetric unit of the structure, is related to the other half by a center of symmetry. Each V^V^ atom is six-coordinated by two strongly (O15, O16) and one weakly (O15^i^) associated oxide groups and by the tridentate Schiff base ligand, *viz*. a phenolate O atom (O7), a secondary amine N atom (N12), both occupying the axial positions, and an imine N atom (N9) (Fig. 1). The geometry about the V atom is distorted octahedral. The V14 ═O16 bond length of 1.612 (2) Å (Table 1) compares well with the distances between V and the doubly bonded O atoms (Romanowski *et al.*, 2008; Root *et al.*, 1993). The V14, O15, V14^i^, O15^i^ atoms are situated at vertices of a parallelogram with the acute O15—V14—O15^i^ angle of 78.64 (8)° [symmetry code: (i) -x, -y+2, -z]. The five-membered ring comprising the ethylenediamine moiety exhibits twofold disorder. The C10 and C11 atoms are disordered over two sites, with occupancy factors of 0.776 (14) and 0.224 (14) for C10A/C11A and C10B/C11B, respectively. The five-membered chelate ring defined by V14, N9, C10A, C11A, N12 adopts an envelope conformation on C10A, with *P* = 244.0 (3)° and τ_(_*_M_*_)_ = 54.9 (4)° for reference bond V14—N9 (Rao *et al.*, 1981) and the ring formed by V14, N9, C10B, C11B, N12 takes the envelope conformation on C11B, with P = 81.8 (7)° and τ_(_*_M_*_)_ = 62.3 (9)° for reference bond V14—N9 (Fig. 1).

In the crystal structure, the dimers are linked through C—H···O hydrogen bonds (Table 1), forming columns along the *a*-axis. There are C—H···π interactions (Fig. 2), involving minor disordered C11B atom [C11B···Cg1^iii^ = 3.47 (2), H11C···Cg1^iii^ = 2.82 Å; Cg1 = centroid of the ring C1–C6; symmetry code: (iii) x, 3/2-y, -1/2+z].

### Experimental

The title compound was obtained in a template/complexation reaction, which was described earlier (Kwiatkowski *et al.*, 2003). A solution of *N*-methylethylenediamine (1 mmol) in absolute EtOH (10 ml) was added under stirring to a freshly filtered solution of vanadium(V) oxytriethoxide (1 mmol) in absolute EtOH (50 ml), producing a yellow suspension of the intermediate. Salicylaldehyde (1 mmol) dissolved in absolute EtOH was added to the aforementioned suspension. After refluxing (70 ml) of the resulting mixture for 2 h and its cooling to room temperature, the separated solids were filtered off, washed several times with EtOH, recrystallized from DMSO-EtOH mixture and dried over molecular sieves.

### Refinement

All H atoms were positioned geometrically and refined using a riding model, with C—H = 0.93 (CH), 0.97 (CH_2_) and 0.96 (CH_3_)Å and N—H = 0.91 Å, and with *U*_iso_(H) = 1.2 (or 1.5 for methyl)*U*_eq_(C,N). The occupancy ratio was determined by isotropic refinement for the disordered site and was refined freely. The minor disordered sites were refined isotropically.

### Crystal data

**Table:**

[V_2_(C_10_H_13_N_2_O)_2_O_4_]	*F*_000_ = 536
*M**_r_* = 520.33	*D*_x_ = 1.557 Mg m^−^^3^
Monoclinic, *P*2_1_/*c*	Mo *K*α radiation λ = 0.71073 Å
Hall symbol: -P 2ybc	Cell parameters from 1960 reflections
*a* = 6.6801 (2) Å	θ = 3.1–25.1º
*b* = 11.9955 (6) Å	µ = 0.89 mm^−^^1^
*c* = 13.8643 (7) Å	*T* = 295 (2) K
β = 92.156 (4)º	Needle, yellow
*V* = 1110.18 (9) Å^3^	0.6 × 0.1 × 0.1 mm
*Z* = 2	

### Data collection

**Table:**

Oxford Diffraction Ruby CCD diffractometer	1960 independent reflections
Radiation source: Enhance (Mo) X-ray Source	1288 reflections with *I* > 2σ(*I*)
Monochromator: graphite	*R*_int_ = 0.050
Detector resolution: 10.4002 pixels mm^-1^	θ_max_ = 25.1º
*T* = 295(2) K	θ_min_ = 3.1º
ω scans	*h* = −7→7
Absorption correction: multi-scan(CrysAlis RED; Oxford Diffraction, 2006)	*k* = −13→14
*T*_min_ = 0.532, *T*_max_ = 0.915	*l* = −13→16
6336 measured reflections	

### Refinement

**Table:**

Refinement on *F*^2^	Secondary atom site location: difference Fourier map
Least-squares matrix: full	Hydrogen site location: inferred from neighbouring sites
*R*[*F*^2^ > 2σ(*F*^2^)] = 0.044	H-atom parameters constrained
*wR*(*F*^2^) = 0.106	*w* = 1/[σ^2^(*F*_o_^2^) + (0.064*P*)^2^] where *P* = (*F*_o_^2^ + 2*F*_c_^2^)/3
*S* = 0.90	(Δ/σ)_max_ < 0.001
1960 reflections	Δρ_max_ = 0.36 e Å^−^^3^
155 parameters	Δρ_min_ = −0.39 e Å^−^^3^
Primary atom site location: structure-invariant direct methods	Extinction correction: none

### Fractional atomic coordinates and isotropic or equivalent isotropic displacement parameters (Å^2^)

**Table:**

	*x*	*y*	*z*	*U*_iso_*/*U*_eq_	Occ. (<1)
C1	0.2360 (4)	0.9625 (3)	0.2273 (2)	0.0439 (8)	
C2	0.4291 (4)	0.9220 (3)	0.2071 (2)	0.0463 (9)	
C3	0.5887 (5)	0.9466 (4)	0.2713 (3)	0.0609 (10)	
H3A	0.7156	0.9199	0.2583	0.073*	
C4	0.5646 (6)	1.0085 (4)	0.3528 (3)	0.0739 (12)	
H4A	0.6735	1.0239	0.3945	0.089*	
C5	0.3749 (6)	1.0482 (4)	0.3724 (3)	0.0696 (11)	
H5A	0.3562	1.0900	0.4278	0.084*	
C6	0.2169 (5)	1.0261 (3)	0.3110 (2)	0.0553 (9)	
H6A	0.0916	1.0542	0.3250	0.066*	
O7	0.0760 (3)	0.9451 (2)	0.16978 (15)	0.0495 (6)	
C8	0.4690 (5)	0.8585 (3)	0.1220 (3)	0.0505 (9)	
H8A	0.5981	0.8309	0.1163	0.061*	
N9	0.3417 (4)	0.8368 (3)	0.0538 (2)	0.0514 (8)	
C10A	0.3951 (9)	0.7645 (7)	−0.0286 (4)	0.0583 (19)	0.776 (14)
H10A	0.5393	0.7566	−0.0312	0.070*	0.776 (14)
H10B	0.3361	0.6911	−0.0226	0.070*	0.776 (14)
C10B	0.442 (3)	0.820 (2)	−0.0377 (13)	0.042 (6)*	0.224 (14)
H10C	0.5616	0.7741	−0.0293	0.050*	0.224 (14)
H10D	0.4755	0.8895	−0.0683	0.050*	0.224 (14)
C11A	0.3133 (8)	0.8217 (7)	−0.1157 (4)	0.058 (2)	0.776 (14)
H11A	0.3336	0.7765	−0.1726	0.070*	0.776 (14)
H11B	0.3797	0.8928	−0.1239	0.070*	0.776 (14)
C11B	0.272 (2)	0.758 (2)	−0.0932 (14)	0.044 (6)*	0.224 (14)
H11C	0.3148	0.7358	−0.1566	0.053*	0.224 (14)
H11D	0.2347	0.6911	−0.0584	0.053*	0.224 (14)
N12	0.0951 (4)	0.8389 (3)	−0.1021 (2)	0.0504 (7)	
H12A	0.0674	0.9027	−0.1355	0.060*	
C13	−0.0425 (6)	0.7569 (4)	−0.1475 (3)	0.0758 (12)	
H13A	−0.0093	0.7463	−0.2136	0.114*	
H13B	−0.1777	0.7836	−0.1448	0.114*	
H13C	−0.0305	0.6872	−0.1137	0.114*	
V14	0.02849 (7)	0.88119 (5)	0.04358 (4)	0.0417 (2)	
O15	−0.1739 (3)	0.95553 (19)	0.00898 (15)	0.0437 (6)	
O16	−0.0525 (3)	0.7584 (2)	0.06852 (19)	0.0629 (7)	

### Atomic displacement parameters (Å^2^)

**Table:**

	*U*^11^	*U*^22^	*U*^33^	*U*^12^	*U*^13^	*U*^23^
C1	0.0506 (18)	0.043 (2)	0.0391 (19)	0.0011 (16)	0.0086 (15)	0.0132 (17)
C2	0.0465 (18)	0.046 (2)	0.047 (2)	0.0026 (16)	0.0079 (16)	0.0110 (17)
C3	0.0506 (19)	0.066 (3)	0.066 (3)	0.0006 (19)	−0.0012 (18)	0.007 (2)
C4	0.061 (2)	0.087 (3)	0.073 (3)	−0.002 (2)	−0.015 (2)	−0.003 (3)
C5	0.084 (3)	0.069 (3)	0.055 (2)	−0.004 (2)	−0.003 (2)	−0.008 (2)
C6	0.063 (2)	0.052 (2)	0.051 (2)	0.0049 (19)	0.0067 (17)	0.005 (2)
O7	0.0427 (11)	0.0664 (17)	0.0397 (13)	0.0116 (12)	0.0066 (10)	0.0001 (12)
C8	0.0410 (17)	0.056 (2)	0.056 (2)	0.0101 (16)	0.0118 (17)	0.0117 (19)
N9	0.0452 (14)	0.065 (2)	0.0452 (17)	0.0181 (14)	0.0153 (14)	0.0048 (16)
C10A	0.050 (3)	0.055 (4)	0.071 (4)	0.015 (3)	0.024 (3)	−0.004 (3)
C11A	0.068 (3)	0.058 (5)	0.050 (3)	0.014 (3)	0.027 (2)	−0.001 (3)
N12	0.0481 (14)	0.0490 (18)	0.0544 (18)	0.0095 (14)	0.0058 (13)	−0.0125 (15)
C13	0.094 (3)	0.063 (3)	0.070 (3)	−0.013 (2)	−0.005 (2)	−0.016 (2)
V14	0.0397 (3)	0.0394 (4)	0.0468 (4)	0.0031 (3)	0.0116 (2)	0.0003 (3)
O15	0.0386 (11)	0.0426 (14)	0.0505 (13)	0.0024 (10)	0.0085 (9)	−0.0031 (11)
O16	0.0645 (14)	0.0455 (15)	0.0805 (19)	0.0012 (12)	0.0262 (13)	0.0087 (14)

### Geometric parameters (Å, °)

**Table:**

C1—O7	1.326 (4)	C10B—C11B	1.54 (3)
C1—C6	1.398 (5)	C10B—H10C	0.9700
C1—C2	1.417 (4)	C10B—H10D	0.9700
C2—C3	1.395 (5)	C11A—N12	1.491 (5)
C2—C8	1.437 (5)	C11A—H11A	0.9700
C3—C4	1.367 (5)	C11A—H11B	0.9700
C3—H3A	0.9300	C11B—N12	1.535 (17)
C4—C5	1.390 (5)	C11B—H11C	0.9700
C4—H4A	0.9300	C11B—H11D	0.9700
C5—C6	1.357 (5)	N12—C13	1.471 (4)
C5—H5A	0.9300	N12—V14	2.146 (3)
C6—H6A	0.9300	N12—H12A	0.9100
O7—V14	1.926 (2)	C13—H13A	0.9600
C8—N9	1.275 (4)	C13—H13B	0.9600
C8—H8A	0.9300	C13—H13C	0.9600
N9—C10B	1.472 (18)	V14—O16	1.612 (2)
N9—C10A	1.489 (6)	V14—O15	1.674 (2)
N9—V14	2.158 (3)	V14—O15^i^	2.316 (2)
C10A—C11A	1.476 (9)	V14—V14^i^	3.1136 (11)
C10A—H10A	0.9700	O15—V14^i^	2.316 (2)
C10A—H10B	0.9700		
			
O7—C1—C6	119.2 (3)	H11A—C11A—H11B	108.5
O7—C1—C2	123.1 (3)	N12—C11B—C10B	106.6 (16)
C6—C1—C2	117.6 (3)	N12—C11B—H11C	110.4
C3—C2—C1	118.8 (3)	C10B—C11B—H11C	110.4
C3—C2—C8	118.4 (3)	N12—C11B—H11D	110.4
C1—C2—C8	122.8 (3)	C10B—C11B—H11D	110.4
C4—C3—C2	122.1 (3)	H11C—C11B—H11D	108.6
C4—C3—H3A	118.9	C13—N12—C11A	116.9 (4)
C2—C3—H3A	118.9	C13—N12—C11B	94.5 (8)
C3—C4—C5	118.9 (4)	C13—N12—V14	114.3 (2)
C3—C4—H4A	120.5	C11A—N12—V14	112.9 (2)
C5—C4—H4A	120.5	C11B—N12—V14	105.1 (7)
C6—C5—C4	120.3 (4)	C13—N12—H12A	103.5
C6—C5—H5A	119.9	C11A—N12—H12A	103.5
C4—C5—H5A	119.9	C11B—N12—H12A	135.8
C5—C6—C1	122.3 (3)	V14—N12—H12A	103.5
C5—C6—H6A	118.9	N12—C13—H13A	109.5
C1—C6—H6A	118.9	N12—C13—H13B	109.5
C1—O7—V14	135.30 (19)	H13A—C13—H13B	109.5
N9—C8—C2	125.3 (3)	N12—C13—H13C	109.5
N9—C8—H8A	117.4	H13A—C13—H13C	109.5
C2—C8—H8A	117.4	H13B—C13—H13C	109.5
C8—N9—C10B	110.8 (7)	O16—V14—O15	105.93 (11)
C8—N9—C10A	121.1 (3)	O16—V14—O7	102.36 (12)
C8—N9—V14	128.1 (2)	O15—V14—O7	98.73 (9)
C10B—N9—V14	116.8 (7)	O16—V14—N12	93.95 (13)
C10A—N9—V14	110.7 (3)	O15—V14—N12	92.87 (10)
C11A—C10A—N9	105.4 (5)	O7—V14—N12	156.46 (10)
C11A—C10A—H10A	110.7	O16—V14—N9	95.31 (12)
N9—C10A—H10A	110.7	O15—V14—N9	156.99 (11)
C11A—C10A—H10B	110.7	O7—V14—N9	84.96 (10)
N9—C10A—H10B	110.7	N12—V14—N9	76.64 (10)
H10A—C10A—H10B	108.8	O16—V14—O15^i^	171.43 (10)
N9—C10B—C11B	98.5 (15)	O15—V14—O15^i^	78.64 (8)
N9—C10B—H10C	112.1	O7—V14—O15^i^	83.83 (9)
C11B—C10B—H10C	112.1	N12—V14—O15^i^	78.44 (10)
N9—C10B—H10D	112.1	N9—V14—O15^i^	79.20 (9)
C11B—C10B—H10D	112.1	O16—V14—V14^i^	152.02 (10)
H10C—C10B—H10D	109.7	O15—V14—V14^i^	46.82 (7)
C10A—C11A—N12	107.1 (5)	O7—V14—V14^i^	90.10 (8)
C10A—C11A—H11A	110.3	N12—V14—V14^i^	82.99 (9)
N12—C11A—H11A	110.3	N9—V14—V14^i^	110.83 (8)
C10A—C11A—H11B	110.3	O15^i^—V14—V14^i^	31.82 (5)
N12—C11A—H11B	110.3	V14—O15—V14^i^	101.36 (8)
			
O7—C1—C2—C3	178.8 (3)	C11B—N12—V14—O16	−68.6 (10)
C6—C1—C2—C3	0.5 (5)	C13—N12—V14—O15	−72.6 (3)
O7—C1—C2—C8	0.1 (5)	C11A—N12—V14—O15	150.5 (4)
C6—C1—C2—C8	−178.2 (3)	C11B—N12—V14—O15	−174.8 (10)
C1—C2—C3—C4	−0.2 (6)	C13—N12—V14—O7	167.7 (3)
C8—C2—C3—C4	178.6 (4)	C11A—N12—V14—O7	30.8 (5)
C2—C3—C4—C5	0.1 (7)	C11B—N12—V14—O7	65.5 (10)
C3—C4—C5—C6	−0.5 (6)	C13—N12—V14—N9	128.1 (3)
C4—C5—C6—C1	0.8 (6)	C11A—N12—V14—N9	−8.8 (4)
O7—C1—C6—C5	−179.2 (3)	C11B—N12—V14—N9	26.0 (10)
C2—C1—C6—C5	−0.8 (5)	C13—N12—V14—O15^i^	−150.4 (3)
C6—C1—O7—V14	171.4 (2)	C11A—N12—V14—O15^i^	72.7 (4)
C2—C1—O7—V14	−6.9 (5)	C11B—N12—V14—O15^i^	107.4 (10)
C3—C2—C8—N9	−174.6 (4)	C13—N12—V14—V14^i^	−118.5 (2)
C1—C2—C8—N9	4.1 (6)	C11A—N12—V14—V14^i^	104.6 (4)
C2—C8—N9—C10B	153.2 (11)	C11B—N12—V14—V14^i^	139.4 (10)
C2—C8—N9—C10A	−176.4 (5)	C8—N9—V14—O16	−104.1 (3)
C2—C8—N9—V14	−2.2 (5)	C10B—N9—V14—O16	101.9 (12)
C8—N9—C10A—C11A	−135.7 (5)	C10A—N9—V14—O16	70.7 (4)
C10B—N9—C10A—C11A	−59.1 (15)	C8—N9—V14—O15	98.4 (4)
V14—N9—C10A—C11A	49.1 (7)	C10B—N9—V14—O15	−55.7 (12)
C8—N9—C10B—C11B	161.4 (12)	C10A—N9—V14—O15	−86.8 (5)
C10A—N9—C10B—C11B	44.5 (16)	C8—N9—V14—O7	−2.1 (3)
V14—N9—C10B—C11B	−40 (2)	C10B—N9—V14—O7	−156.2 (12)
N9—C10A—C11A—N12	−55.7 (8)	C10A—N9—V14—O7	172.7 (4)
N9—C10B—C11B—N12	63 (2)	C8—N9—V14—N12	163.1 (3)
C10A—C11A—N12—C13	−97.6 (6)	C10B—N9—V14—N12	9.0 (11)
C10A—C11A—N12—C11B	−44.7 (12)	C10A—N9—V14—N12	−22.1 (4)
C10A—C11A—N12—V14	38.2 (8)	C8—N9—V14—O15^i^	82.6 (3)
C10B—C11B—N12—C13	−175.1 (16)	C10B—N9—V14—O15^i^	−71.5 (11)
C10B—C11B—N12—C11A	50.5 (15)	C10A—N9—V14—O15^i^	−102.7 (4)
C10B—C11B—N12—V14	−58.4 (19)	C8—N9—V14—V14^i^	86.1 (3)
C1—O7—V14—O16	101.1 (3)	C10B—N9—V14—V14^i^	−68.0 (12)
C1—O7—V14—O15	−150.4 (3)	C10A—N9—V14—V14^i^	−99.1 (4)
C1—O7—V14—N12	−31.7 (5)	O16—V14—O15—V14^i^	−172.54 (11)
C1—O7—V14—N9	6.8 (3)	O7—V14—O15—V14^i^	81.85 (10)
C1—O7—V14—O15^i^	−72.9 (3)	N12—V14—O15—V14^i^	−77.60 (10)
C1—O7—V14—V14^i^	−104.1 (3)	N9—V14—O15—V14^i^	−15.9 (3)
C13—N12—V14—O16	33.6 (3)	O15^i^—V14—O15—V14^i^	0.0
C11A—N12—V14—O16	−103.3 (4)		

Symmetry codes: (i) −*x*, −*y*+2, −*z*.

### Hydrogen-bond geometry (Å, °)

**Table:**

*D*—H···*A*	*D*—H	H···*A*	*D*···*A*	*D*—H···*A*
C8—H8A···O16^ii^	0.93	2.60	3.520 (4)	170
C11B—H11C···Cg1^iii^^iii^	0.97	2.82	3.47 (2)	124

Symmetry codes:  (ii) *x*+1, *y*, *z*; (iii) *x*, −*y*+3/2, *z*−1/2.
